# Optimizing irrigation and nitrogen application strategies to improve sunflower yield and resource use efficiency in a cold and arid oasis region of Northwest China

**DOI:** 10.3389/fpls.2024.1429548

**Published:** 2024-08-30

**Authors:** Xietian Chen, Hengjia Zhang, Shouchao Yu, Chenli Zhou, Anguo Teng, Lian Lei, Yuchun Ba, Fuqiang Li

**Affiliations:** ^1^ College of Water Conservancy and Hydropower Engineering, Gansu Agricultural University, Lanzhou, China; ^2^ College of Agriculture and Biology, Liaocheng University, Liaocheng, China; ^3^ Yimin Irrigation Experimental Station, Hongshui River Management Office, Zhangye, China

**Keywords:** dry matter accumulation, multi-objective optimization, sunflower yield, water and nitrogen use efficiency, drip fertigation

## Abstract

In arid regions, water scarcity, land degradation and groundwater pollution caused by excessive fertilization are the main constraints to sustainable agricultural production. Optimizing irrigation and fertilizer management regime is an effective means of improving crop water and fertilizer productivity as well as reducing negative impacts on the ecosystem. In order to investigate the effects of different irrigation and nitrogen (N) fertilizer rates on sunflower growth, yield, and water and N use efficiency, and to determine the optimal water and N management strategy, a two-year (2021 and 2022) field experiment with under-mulched drip irrigation was conducted in the Hexi Oasis area of Northwest China. The experiment design comprised three irrigation levels (W1, 55%−65% F_C,_ where F_C_ represents field water capacity; W2, 65%−75% F_C_; W3, 75%−85% F_C_) and three N application levels (N1, 120 kg ha^–1^; N2, 180 kg ha^–1^; N3, 240 kg ha^–1^), resulting in a total of nine treatments. The findings indicated that increasing irrigation and N application rates led to improvements in leaf area index (15.39%−66.14%), dry matter accumulation (11.43%−53.15%), water consumption (ET, 1.63%−42.90%) and sunflower yield (6.85%−36.42%), in comparison to the moderate water deficit and low N application (W1N1) treatment. However, excess water and N inputs did not produce greater yield gains and significantly decreased both water use efficiency (WUE) and nitrogen partial factor productivity (NPFP). Additionally, a multiple regression model was developed with ET and N application as explanatory variables and yield, WUE and NPFP as response variables. The results based on the regression model combined with spatial analysis showed that an ET range of 334.3−348.7 mm and N application rate of 160.9−175.3 kg ha^–1^ achieved an optimal balance between the multiple production objectives: yield, WUE and NPFP. Among the different irrigation and N management strategies we evaluated, we found that W2N2 (65%−75% F_C_ and 180 kg N ha^–1^) was the most fruitful considering yield, resource use efficiency, etc. This result can serve as a theoretical reference for developing appropriate irrigation and N fertilization regimes for sunflower cultivation in the oasis agricultural area of northwest China.

## 1 Introduction

The Hexi Oasis agricultural area is recognized as a critical commercial grain base and a hub for cash crop production in Northwest China, earning its reputation as the “Northwest Granary” (Wu et al., 2021). The agricultural output in this region is paramount to ensuring food security and sustained economic progress in Northwest China (Chen et al., 2022). However, the arid climate, water scarcity and ecological fragility have seriously constrained the sustainable development of agriculture in this region (Li et al., 2015; Wang et al., 2023a). Over the past three decades, the continuous expansion of the oasis area in Hexi has resulted in a serious imbalance between water and land resources, further intensifying the agricultural water crisis (Sun et al., 2021). In addition, the pursuit of high crop yields has led to widespread excessive irrigation and fertilization practices in the Hexi Oasis region (Pan et al., 2024). This traditional “high yield − high water – high fertilizer” production model not only contributes to inefficient water and fertilizer use but also raises concerns about environmental consequences (Campbell et al., 2011; Lu et al., 2021a; Wang et al., 2023b; Zhang et al., 2016). These consequences include nitrogen leaching losses, groundwater pollution and soil quality degradation. Therefore, to achieve sustainable agricultural production and establish a positive feedback loop for the ecological environment in the Hexi Oasis area, a two-pronged approach is essential: the rational allocation of regional land and water resources, and optimization of irrigation and fertilization management strategies.

As one of the four most important oilseed crops in the world, sunflower (*Helianthus annuus L.*) is widely planted in different countries and regions due to its excellent adaptability to various environments and climates (Sinha et al., 2017; Stone et al., 2002). In 2020 alone, China planted 900,000 ha of sunflowers—representing approximately 3% of the world’s total—and harvested a yield of 2.38 million tons, rendering it the fifth-largest producer globally (Zhang et al., 2023). In China, most sunflower cultivation occurs in the arid and semi-arid northern regions, with the Hexi Oasis irrigated area standing out as a major sunflower production center. In recent years, however, traditional irrigation practices (e.g. furrow and flood irrigation) have still been used for sunflower production in this area, resulting in serious waste and inefficient use of water resources (Wang et al., 2021a). Thankfully, research has indicated that drip fertigation technology can well coordinate the supply relationship between water and nutrients, thereby promoting the efficient use of water and fertilizer (Yan et al., 2019). This technology delivers water and fertilizer solution to the crop root system in a uniform, continuous and accurate manner through a pipeline system, thus avoiding nutrient loss and reducing the risk of soil environmental pollution (Li et al., 2022a; Ma et al., 2022). While drip fertigation is slowly being integrated into sunflower cultivation, developing sustainable and scientifically-backed drip irrigation and fertilizer management regimes remains a major challenge for agricultural production in the Hexi Oasis region.

As a fundamental building block of nucleic acids and proteins, nitrogen (N) is critical for maintaining plant life activities and crop yield formation (Long et al., 2021; Sinclair and Rufty, 2012). In irrigated agriculture, optimizing both water and N management is crucial for achieving high crop yields while promoting resource use efficiency and environmental sustainability (Ballester et al., 2021; Liu et al., 2019). Studies have demonstrated significant interactive and complementary effects between water and N (Sheshbahreh et al., 2019). While water can enhance N use efficiency, N, accordingly, affects plant growth and development, thereby affecting water uptake by crops (Gao et al., 2023; Wang et al., 2014a). However, the effects of water and N supply on crop growth and yield can be complex, resulting in either synergistic or antagonistic interactions depending on the specific input levels of both (Feng et al., 2023; Wang et al., 2016). For instance, Wang et al. (2021a) observed that under water stress combined with high N fertilization, excess N accumulated in the soil. This accumulation led to increased soil solution concentrations, hindering both water and nutrient uptake by sunflowers; whereas, excessive irrigation can lead to N leaching into deeper soil layers, depleting N levels in the root zone and potentially contaminating the groundwater resources. Kiani et al. (2016) highlighted that under severe water deficit conditions, simply increasing N fertilizer application is not an effective strategy for sunflowers. The optimum N application depends on various factors such as the source of N fertilizer and irrigation levels. Therefore, to develop effective water and N management strategies for sustainable and efficient crop production, it is essential to conduct field experiments that investigate how sunflower growth and yield to different irrigation and N application rates under specific climatic conditions.

Multiple regression modeling has become a commonly applied method for determining the optimal irrigation and fertilization practices in recent years (Hao et al., 2022; He et al., 2023). For instance, Li et al. (2022b) devised a quadratic equation to represent the relationship between economic efficiency, irrigation amount, and N application rate. Utilizing this equation, they determined the optimal irrigation amount to be 360 mm with a corresponding N fertilizer rate of 225 kg ha^–1^ for sunflower production in the Hetao irrigation area of Inner Mongolia, China. Similarly, Zhang et al. (2023) employed a regression equation with sunflower yield as the dependent variable to determine the optimal irrigation and N application rates for sunflower production in the arid region of northern China. Their findings indicated that an irrigation amount of 159.2–177.1 mm and an N application rate of 166.0–218.3 kg ha^–1^ were most suitable. However, relying solely on a single indicator such as crop yield for evaluating irrigation and fertilization practices may not be sufficient to optimize other crucial production factors, such as water and N use efficiency (Xing et al., 2015). A more comprehensive approach involves considering multiple objectives simultaneously. To address this, previous studies have proposed constructing multiple binary quadratic models, each representing a specific objective. By projecting the resulting response surfaces utilizing spatial analysis, researchers can identify areas of overlap where acceptable ranges for each indicator intersect. This overlapping region represents the optima range for both water and N application (Thompson et al., 2000). This method has been successfully applied to establish multi-objective water and N management strategies for various crops, including tomato (Xing et al., 2015), maize (Feng et al., 2023), winter wheat (Gao et al., 2023) and cotton (Wang et al., 2018). Notwithstanding the success of this approach, there is limited research on optimizing water and N management strategies for sunflower production in oasis irrigated areas.

This research operates under the hypothesis that the current water and N application strategies employed for sunflower cultivation in the Hexi Oasis region of Northwest China are not conducive to maximizing production returns. Therefore, this study aims to propose a high-yielding, efficient and environmentally sound water and N management strategy based on a multi-objective regression model coupled with spatial analysis derived from a field trial. The specific objectives of this study are: (1) to investigate the interactive effects of different irrigation and N application rates on leaf area index (LAI), aboveground biomass, water consumption (ET), yield, and water and N use efficiency of sunflower; (2) to develop a multiple regression model utilizing ET and N application as independent variables and yield, water and N use efficiency as dependent variables; and (3) to determine the optimal water and N management strategies for sunflower in arid areas based on the regression equations and spatial analysis. This study aims to offer theoretical guidance for the efficient management of water and fertilizer in sunflower production in oasis irrigated areas in Northwest China.

## 2 Materials and methods

### 2.1 Experimental site and climatic conditions

**Figure 1:**
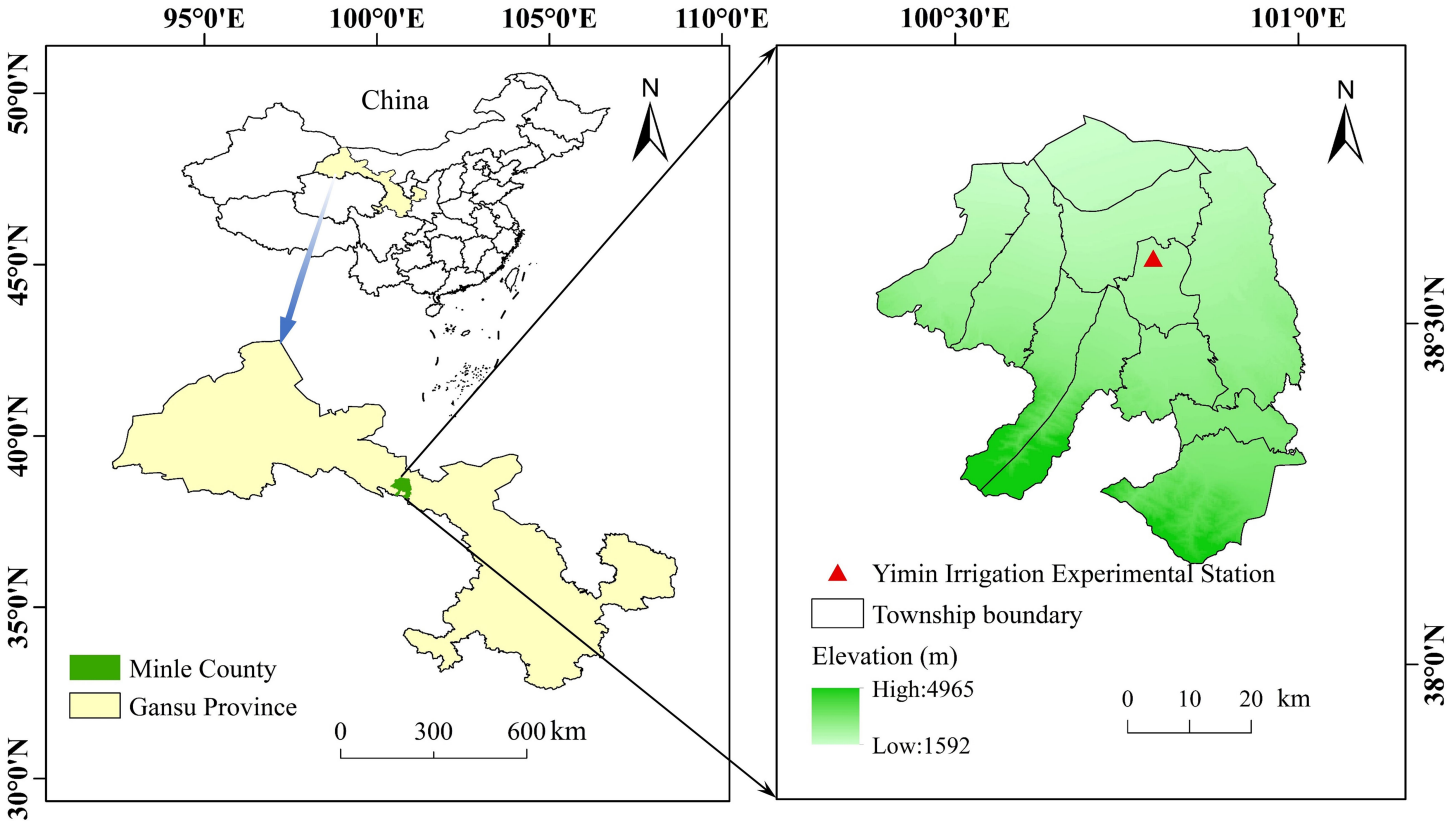
Location of the experimental site.

**Figure 2:**
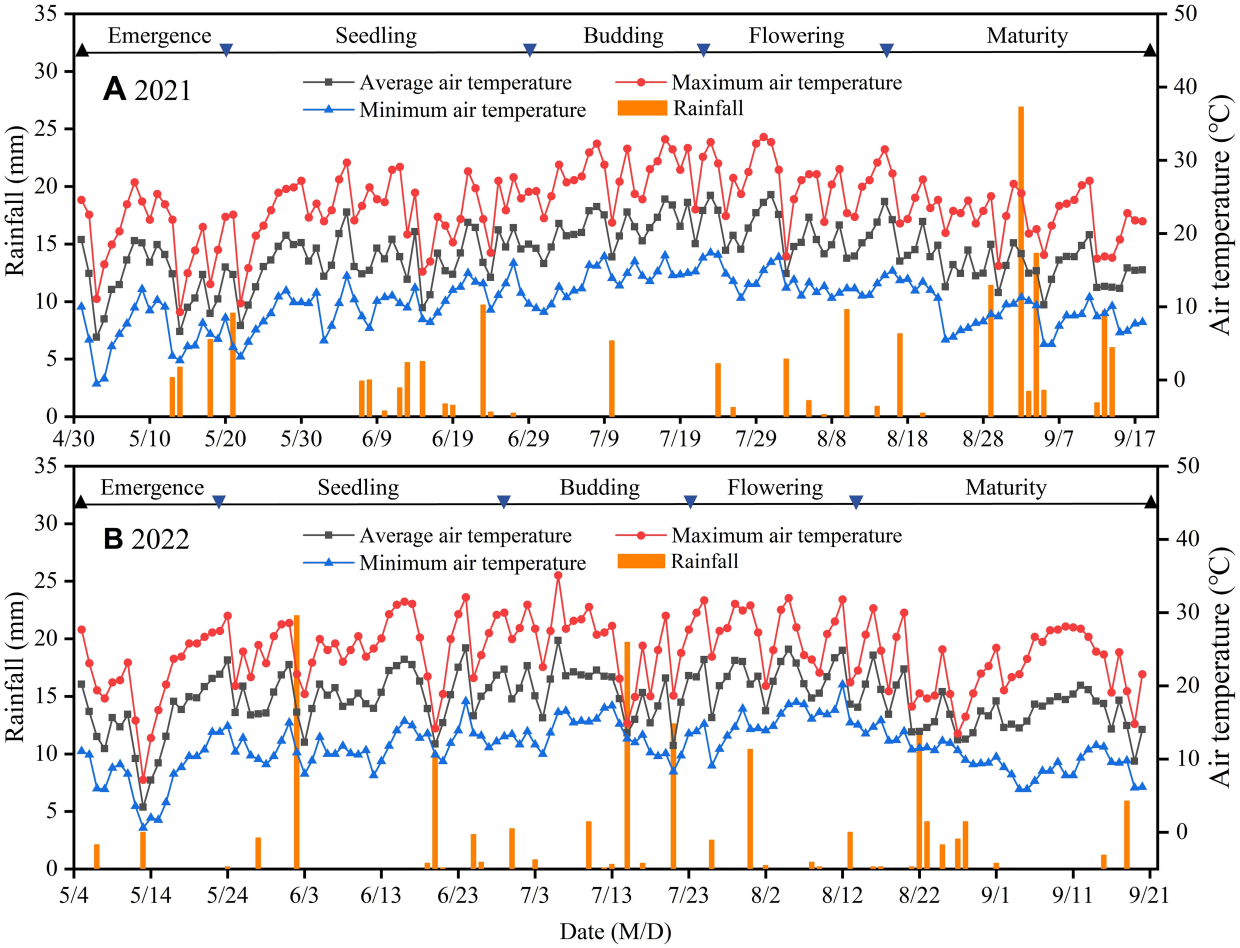
Daily air temperature and rainfall at the experimental site during the sunflower growing season in 2021 **(A)** and 2022 **(B)**.

A 2-year (2021 and 2022) field trial was performed at the Yimin Irrigation Experimental Station (100°47′ E, 38°35′ N, 1976.9 m a.s.l.) in Minle County, Gansu Province, Northwest China (**Figure 1**). The test region is characterized by an arid continental climate, with significant temperature differences between day and night. The region receives sufficient light and heat resources, offering naturally advantageous conditions for agricultural production. However, with average annual rainfall decline below 200 mm and average annual evaporation exceeding 2000 mm, irrigation becomes critical to sustain regular crop production. The study site experiences an average annual temperature of 6.2°C, approximately 3000 h of annual sunshine, and a frost-free period of approximately 140 days. Groundwater, at a depth exceeding 20 m, constitutes a primary source for local irrigation (Wang et al., 2023a). In 2021 and 2022, total rainfall during the sunflower growing season reached 164.4 mm and 136.3 mm, with average air temperatures of 17.30°C and 18.16°C, respectively (**Figure 2**). Additionally, the physical and chemical properties of the 0–60 cm soil profile at the test site were analyzed before sowing, as presented in **Table 1**.

**Table 1:** Soil physical and chemical properties (0–60 cm) at the experimental site.

Soil layer (cm)	Particle composition (%)			Soil texture	Organic matter (g kg^–1^)	Nitrate nitrogen (mg kg^–1^)	Ammonium nitrogen (mg kg^–1^)	Available phosphorus (mg kg^–1^)	Available potassium (mg kg^–1^)	Field capacity(%)	Bulk density (g cm^–3^)	pH
	< 0.002 mm	0.002−0.02 mm	0.02−2 mm									
0−20	11.27	55.45	33.28	Silty loam	10.08	24.74	38.82	27.51	133.79	24.53	1.42	7.9
20−40	13.78	50.47	35.75	Silty loam	9.22	25.17	37.31	20.19	77.59	23.47	1.48	7.7
40−60	12.77	40.68	46.55	loam	8.68	14.27	35.56	16.00	58.48	23.89	1.49	8.0

### 2.2 Experimental design and field management

The experiment was conducted in a completely randomized block design with two factors and three replications. Three irrigation levels were employed: moderate water deficit (W1, 55%–65% F_C_, F_C_ is the field water capacity), mild water deficit (W2, 65%–75% F_C_) and full irrigation (W3, 75%–85% F_C_). The N application rates consisted of three levels: low N fertilization (N1, 120 kg ha^–1^), medium N fertilization (N2, 180 kg ha^–1^) and high N fertilization (N3, 240 kg ha^–1^, which was the local conventional N application rate). The experiment comprised nine treatments across 27 plots, with each plot measuring 27.2 m^2^ (3.4 m×8 m). Adjacent plots were separated by 100 cm wide protected zones and were also fitted with 60 cm deep plastic membranes to prevent horizontal movement of water and N. Each irrigation event was initiated based on soil water content measurements at the planned wetted layer depth (40 cm during the seedling stage and 60 cm at other stage, contingent upon root distribution depth). When the soil water content neared or fell below the designated lower limit, irrigation promptly restored it to the designated upper limit. The calculation method for each irrigation amount is detailed in Wang et al. (2023a). Urea (N, 46%), superphosphate (P_2_O_5_, 12%) and potassium sulphate (K_2_O, 51%) were selected as the sources of N, P and K. Prior to sowing, 150 kg P_2_O_5_ ha^–1^ and 180 kg K_2_O ha^–1^ of phosphorus and potassium fertilizer and 40% N fertilizer were applied as base fertilizer for each treatment, respectively. The remaining N fertilizer was delivered through drip fertigation during the early stages of budding (30%) and flowering (30%). The specific irrigation and N application schemes are shown in **Table 2**.

**Table 2:** Irrigation and nitrogen application schemes for the experiment.

Treatment	Water deficit level (% of field water capacity)	The amount and stage of nitrogen application (kg ha^–1^)			
		Sowing (40%)	Budding (30%)	Flowering (30%)	Total amount
W1N1	55%−65%	48	36	36	120
W1N2	55%−65%	72	54	54	180
W1N3	55%−65%	96	72	72	240
W2N1	65%−75%	48	36	36	120
W2N2	65%−75%	72	54	54	180
W2N3	65%−75%	96	72	72	240
W3N1	75%−85%	48	36	36	120
W3N2	75%−85%	72	54	54	180
W3N3	75%−85%	96	72	72	240

W1, moderate water deficit (55%−65% of field water capacity, F_C_); W2, mild water deficit (65%−75% F_C_); W3, full irrigation (75%−85% F_C_). N1, low nitrogen rate (120 kg ha^–1^); N2, medium nitrogen rate (180 kg ha^–1^); N3, high nitrogen rate (240 kg ha^–1^).

**Figure 3:**
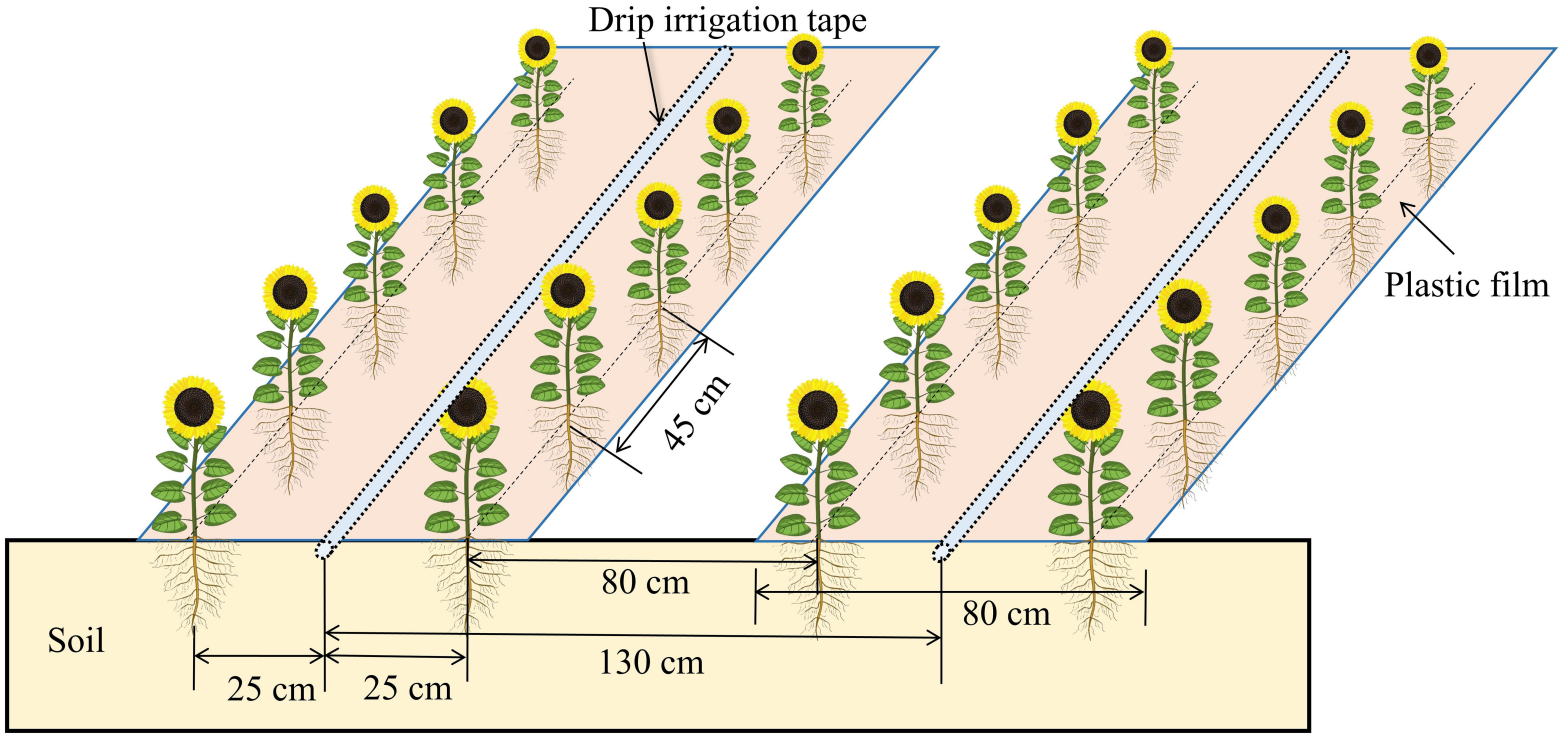
The sunflower planting pattern and the layout of drip irrigation under plastic film.

The sunflower cultivar “JK601” (a widely grown variety in the region) was planted on 1^st^ May 2021 and 5^th^ May 2022, respectively. The harvest dates were 18^th^ September 2021 and 20^th^ September 2022, respectively. Seeds were planted by hand at a depth of 5 cm using a hole seeder. Irrigation was offered through drip tapes beneath plastic film (80 cm wide, 0.01 mm thick). Two rows of sunflowers were planted on each film, with a narrow row spacing of 50 cm, a wide row spacing of 80 cm and a plant spacing of 45 cm. A single irrigation tape (16 mm diameter) was centered under each film. Drippers were positioned every 30 cm along the tape, offering a flow rate of 3.0 L hour^–1^. **Figure 3** illustrates the sunflower planting pattern and the layout of drip irrigation under plastic film.

### 2.3 Measurements, calculations and methodologies

#### 2.3.1 Determination of leaf area index

Five sunflower plants were randomly sampled from each plot at the seedling (S), budding (B), flowering (F), and maturity (M) stages, and the maximum length and width of all leaves were determined using a steel ruler. Leaf area index (LAI) was calculated according to the following formula (Guo et al., 2019):


(1) $$LAI=\frac{0.6564\times NP\times \sum_{i=1}^{n}LL\times LB}{A}$$

where 0.6564 was the conversion factor of sunflower leaf area (Ma et al., 2020); *NP* was the total number of plants in a single plot; *LL* was the maximum leaf length of fully extended leaves of sunflower (cm); *LB* was the maximum leaf width (cm); *n* was the number of leaves in a single plant; *A* was the area of a single plot (cm^2^).

#### 2.3.2 Dry matter accumulation and seed yield

After sunflower reached the S stage, we randomly selected three plants from each plot every 20 days to determine the aboveground dry matter accumulation (DMA). The fresh sunflowers were separated into different organs (stems, leaves and flower discs). Each plant component was initially heated in an oven at 105 °C for 30 minutes, then dried to constant weight at 75°C. To calculate the DMA per hectare, we factored in the average weight of each plant, the sowing density and the seedling emergence rate (Wang et al., 2021b). After seed maturity, ten flower heads were selected from each plot for threshing and natural drying. Subsequently, the 100-seed weight and seed weight per head were determined and converted to yield per hectare.

#### 2.3.3 Water consumption

Sunflower growing season water consumption (ET) was calculated as follows (Lu et al., 2021b):


(2) ET=P+U+I+ΔW−D−R

where *P* is rainfall (mm); *U* is groundwater recharge (mm); *I* is irrigation (mm); *R* and *D* are surface runoff and deep drainage (mm), respectively; and *ΔW* is the change in soil water storage between planting and harvest (mm). In this experiment, the soil water content was always below *F_C_* and the groundwater depth was greater than 20 m. Therefore, the values of *U*, *D*, and *R* were set to zero.

#### 2.3.4 Productivity indicators

Water use efficiency (WUE) and irrigation water use efficiency (IWUE) were calculated as follows (Pan et al., 2024):


(3) $$WUE=\frac{GY}{10\times ET}$$

(4) $$IWUE=\frac{GY}{10\times I}$$

where *GY* represents the grain yield (kg ha^–1^), *ET* denotes the water consumption (mm), and *I* depicts the irrigation amount (mm).

Nitrogen partial factor productivity (NPFP) was calculated by the following formula (Gao et al., 2023):


(5) $$NPFP=\frac{GY}{N_{T}}$$

where *N_T_* represents the total nitrogen applied (kg ha^–1^).

#### 2.3.5 Logistic model

A logistic model was employed to explore the response of DMA to different irrigation and N application rates utilizing days after seedling emergence as the independent variable. The logistic equation is expressed as follows (Ding et al., 2019):


(6) $$y=\frac{k}{1+{\text{ae}}^{-bt}}$$

where *y* is the dry matter accumulation of sunflower (kg ha^–1^), *k* is the maximum dry matter accumulation (kg ha^–1^), *t* is the number of days after emergence (d), and *a* and *b* are parameters to be determined.

Taking the first derivative of the logistic equation gives the cumulative rate equation:


(7) $$y=\frac{{\text{ab}ke}^{-bt}}{{\left(1+{ae}^{-bt}\right)}^{2}}$$

Based on Equations 6 and 7 the values of the relevant characteristic parameters can be calculated as follows:


(8) $$V_{max}=\frac{kb}{4}$$

(9) $$t_{max}=\frac{ln\;a}{b}$$

(10) $$t_{1}=\frac{1}{b}ln\;(\frac{a}{2+\sqrt{3}})$$

(11) $$t_{2}=\frac{1}{b}ln\;(\frac{a}{2-\sqrt{3}})$$

(12) $$\Delta t=t_{2}-t_{1}$$

where *V_max_* (kg ha^–1^ d^–1^) and *t_max_* (d) are the maximum accumulation rate of dry matter and its occurrence time; *t_1_* (d) and *t_2_* (d) are the start and end time of the rapid growth period, respectively; *Δt* (d) is the duration of rapid growth.

#### 2.3.6 Water and nitrogen regression model based on multiple objectives

In this study, the effect of different water and N treatments on multiple target variables were evaluated by establishing a binary quadratic regression equation. The regression equation is expressed as follows (Pan et al., 2024):


(13) $$y=y_{0}+{ax}_{1}+{bx}_{2}+{cx}_{1}x_{2}+{dx}_{1}^{2}+{ex}_{2}^{2}$$

where *y* represents the response variable; *x_1_* and *x_2_* denote water consumption and nitrogen application rate, respectively; *y_0_* depicts a constant term; and *a, b, c, d* and *e* indicate coefficients to be determined.

#### 2.3.7 Data analysis

The data presented in the figures and tables represent the mean of three replicates. Excel 2019 was used for fundamental data organization and calculations. Analysis of variance (ANOVA) and regression equation modeling for each year’s data were performed using SPSS 22.0 (SPSS Inc., Chicago, USA) software. Irrigation (W) and nitrogen application (N) and their interaction (W×N) were considered as fixed factors and replication was considered as a random factor. Duncan’s multiple range test was employed for multiple comparisons of means between treatments at a significance level of P < 0.05. Origin 2021 (OriginLab Inc., USA) was used to generate the figures.

## 3 Results

### 3.1 Leaf area index

**Figure 4:**
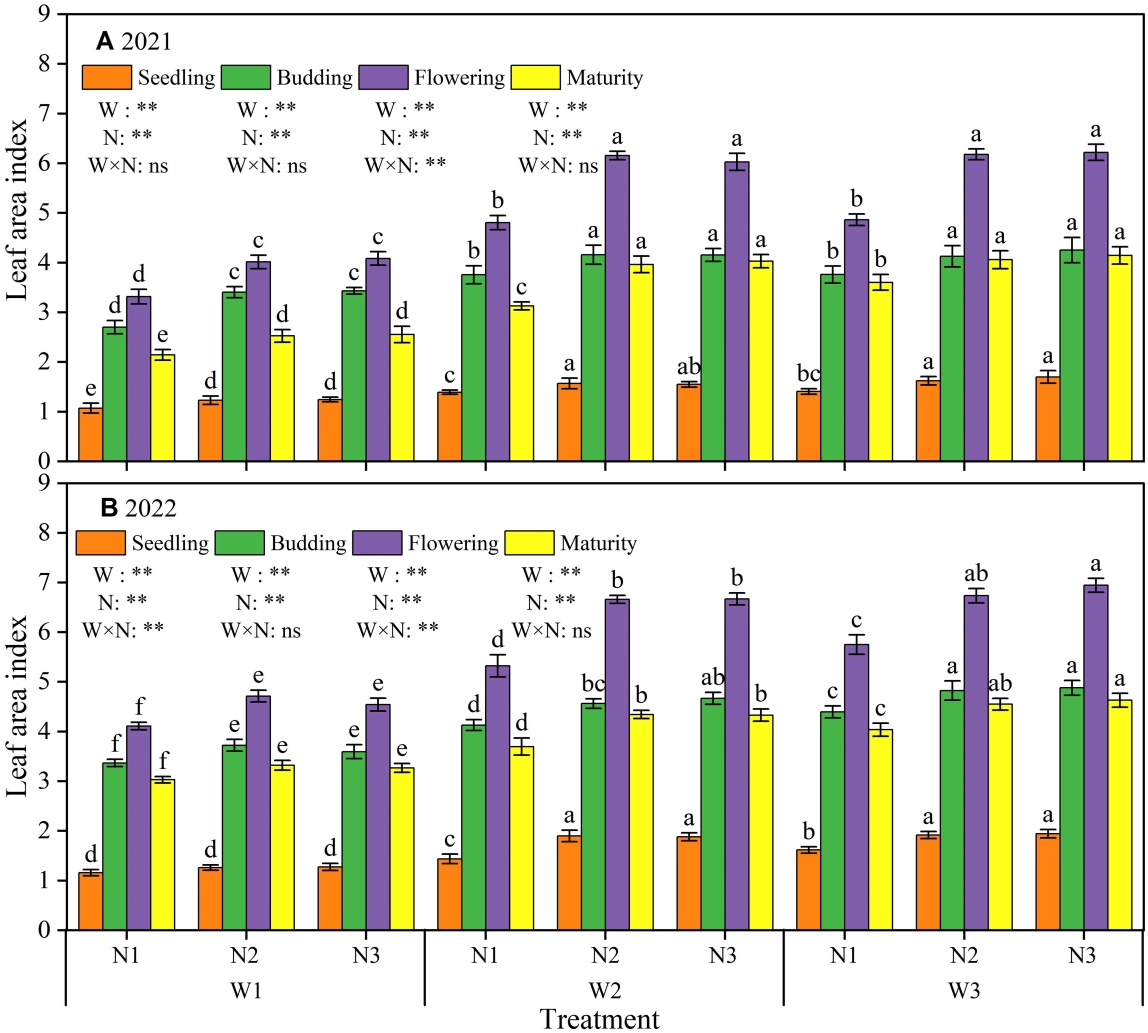
Effect of different irrigation and nitrogen application treatments on leaf area index in 2021 **(A)** and 2022 **(B)**. W1, moderate water deficit (55%−65% of field water capacity, F_C_); W2, mild water deficit (65%−75% F_C_); W3, full irrigation (75%−85% F_C_). N1, low nitrogen rate (120 kg ha^–1^); N2, medium nitrogen rate (180 kg ha^–1^); N3, high nitrogen rate (240 kg ha^–1^). Different lowercase letters above the error bar indicate significant differences between treatments in the same period at the P < 0.05 level. ** indicates a highly significant effect (P < 0.01); and ns indicates a non-significant effect (P > 0.05).

As shown in **Figure 4**, LAI under all treatments exhibited an initial increase followed by a decrease with the progression of growth stages, attaining a peak at the F period. Irrigation and N application treatments had a highly significant (P < 0.01) effect on LAI at each growth stage in both 2021 and 2022. Specifically, LAI was highest in W3N3 and lowest in W1N1 during the entire growing period. In comparison to W3N3, the LAI of W1N1 displayed reductions of 36.90%, 36.46%, 46.65% and 48.29% in 2021 and 40.35%, 31.00%, 40.81% and 33.85% in 2022 for S, B, F and M stages, respectively. Under consistent water deficit conditions, the LAI of N2 and N3 exhibited no significant (P > 0.05) difference, whereas the LAI of N1 was significantly lower than that of N2 and N3. In the concluding M stage, the LAI of N1 reduced by 15.9% and 17.3% in 2021 and by 11.9% and 11.6% in 2022, relative to N2 and N3, respectively. Under conditions of constant N application, LAI declined with increasing water deficit across each growth stage. The LAI of W1 was significantly lower than that of W2 and W3, but the difference in LAI between W2 and W3 was not significant. Relative to W3, the LAI of W1 at the S, B, F and M stages exhibited reductions of 24.86%, 21.41%, 33.85% and 38.83% in 2021, and 32.52%, 24.23%, 31.19% and 26.93% in 2022, respectively. In general, increasing irrigation and N application led to an increase in LAI, but past a specific limit, the effect ceased to be significant.

### 3.2 Dry matter accumulation characteristics

**Figure 5:**
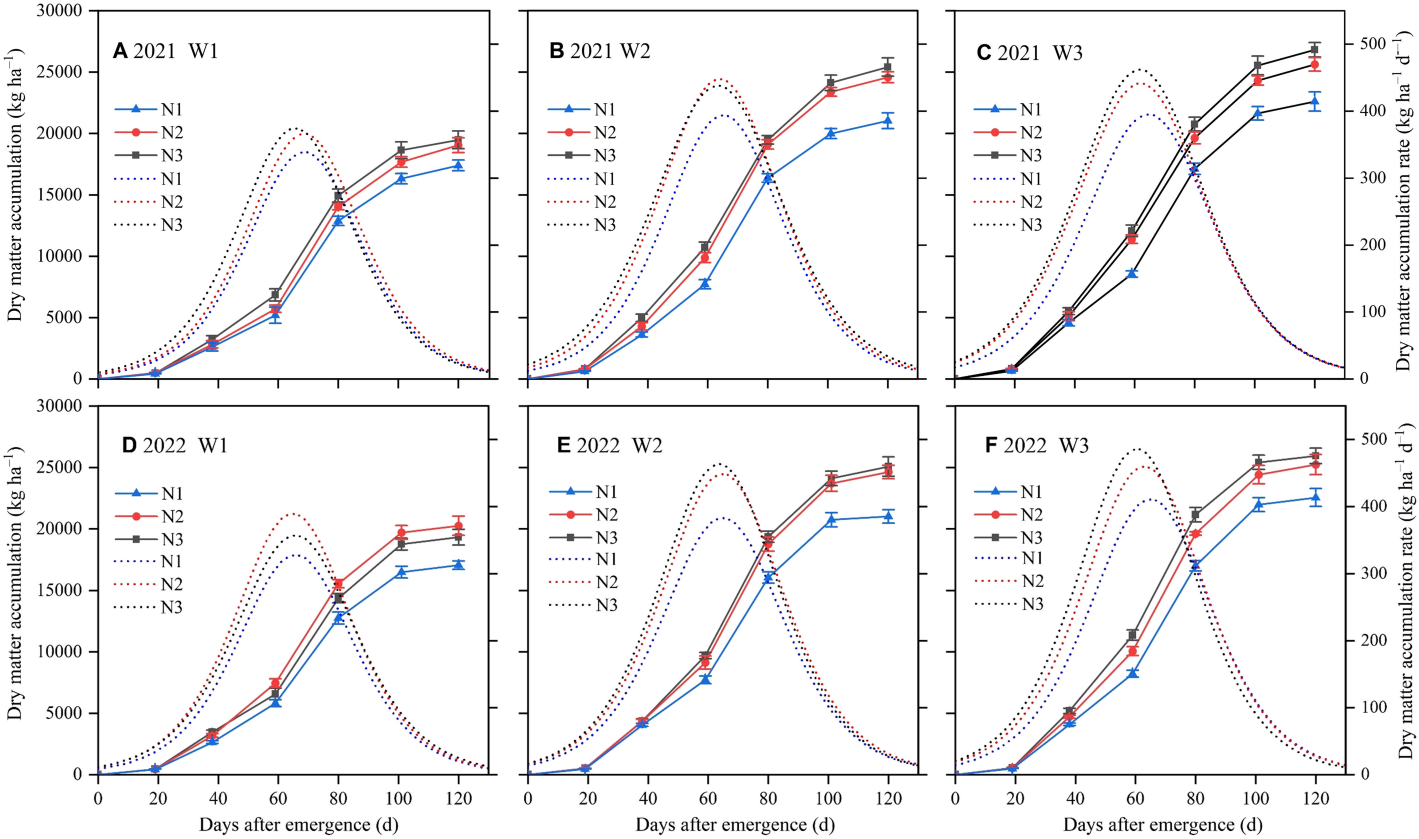
Effects of different irrigation and nitrogen treatments on dry matter accumulation and dry matter accumulation rate in 2021 **(A–C)** and 2022 **(D–F)**. W1, moderate water deficit (55%−65% of field water capacity, F_C_); W2, mild water deficit (65%−75% F_C_); W3, full irrigation (75%−85% F_C_). N1, low nitrogen rate (120 kg ha^–1^); N2, medium nitrogen rate (180 kg ha^–1^); N3, high nitrogen rate (240 kg ha^–1^). The solid and dashed lines represent dry matter accumulation and dry matter accumulation rate, respectively.

The aboveground dry matter (DM) of sunflower under different irrigation and N application treatments steadily accumulated as growth stages progressed, exhibiting a pattern of gradual increase in the early and late stages, with a rapid increase during the middle stage (**Figure 5**). Additionally, significant differences in DM were observed across different irrigation and N application treatments. At consistent irrigation levels, DM increased with increasing N application. In 2021 and 2022, the DM in N1 was lower by 14.87% and 13.83% relative to N2 and by 11.85% and 13.54% relative to N3 in the F stage, respectively. Under the same N application conditions, DM declined with increasing water deficit. In both growing seasons, DM was significantly lower in W1 than in W2 and W3. At the end of the F stage, DM in W1 experienced reductions of 25.47% and 23.18% compared to W3 and 21.28% and 19.91% compared to W2 in both years, respectively. In general, W3N3 yielded the highest DM among all treatments, demonstrating an increase of 54.10% in 2021 and 52.20% in 2022 relative to the lowest W1N1.

The DMA rate and its associated characteristic parameters fitted by the logistic model were shown in **Figure 5** and **Table 3**, respectively. The results indicated that the determination coefficients (R^2^) of the regression models consistently exceeded 0.99, suggesting that the logistic equation effectively modeled the process of DMA in sunflower with the days after seedling emergence. The duration of rapid growth in sunflower DMA across all treatments spanned from 33.77 d to 38.17 d. This duration contracted with decreasing irrigation and N application in 2021, while this pattern was not replicated in 2022. Moreover, the start of the rapid growth period in sunflower DMA occurred earlier with increasing irrigation and N application. In both seasons, the initiation of the rapid growth phase in N3 and N2 was observed on average 2.57 d and 1.23 d earlier than in N1, respectively. Similarly, the beginning of the rapid growth phase in W3 and W2 was on average 4.84 d and 1.90 d earlier than in W1, respectively. With increasing irrigation and N application, the maximum DMA rate in sunflower increased and its appearance was advanced. In 2021 and 2022, the maximum DMA rate exhibited increases of 12.97% and 16.44% in N3 and 11.32% and 15.71% in N2, respectively, compared to N1.

**Table 3:** Logistic model parameters and characteristic values of sunflower dry matter accumulation under different irrigation and nitrogen application treatments.

Year	Treatment	Regression equation	R^2^	*t_1_* (d)	*t_2_* (d)	*t_max_* (d)	*Δt* (d)	*V_max_* (kg ha^–1^ d^–1^)
2021	W1N1	y=17398.01/(1 + 204.06e^–0.078x^)	0.996	51.30	85.07	68.18	33.77	339.26
	W1N2	y=19048.33/(1 + 190.89e^–0.077x^)	0.997	51.10	85.31	68.20	34.21	366.68
	W1N3	y=19478.71/(1 + 151.80e^–0.077x^)	0.997	48.12	82.33	65.23	34.21	374.97
	W2N1	y=21040.56/(1 + 132.60e^–0.075x^)	0.998	47.60	82.72	65.16	35.12	394.51
	W2N2	y=24582.67/(1 + 106.24e^–0.073x^)	0.999	45.87	81.95	63.91	36.08	448.63
	W2N3	y=25419.8/(1 + 79.21e^–0.069x^)	0.998	44.28	82.45	63.36	38.17	438.49
	W3N1	y=22607.74/(1 + 91.64e^–0.070x^)	0.995	45.73	83.36	64.54	37.63	395.64
	W3N2	y=25622.8/(1 + 71.90e^–0.069x^)	0.998	42.71	80.88	61.80	38.17	441.99
	W3N3	y=26809.49/(1 + 70.540e^–0.069x^)	0.998	42.60	80.77	61.68	38.17	462.46
2022	W1N1	y=17049.45/(1 + 157.56e^–0.077x^)	0.996	48.61	82.82	65.71	34.21	328.20
	W1N2	y=20259.61/(1 + 146.96e^–0.077x^)	0.997	47.70	81.91	64.81	34.21	390.00
	W1N3	y=19329.24/(1 + 128.91e^–0.074x^)	0.993	47.87	83.46	65.66	35.59	357.59
	W2N1	y=21019.91/(1 + 113.64e^–0.073x^)	0.992	46.79	82.88	64.84	36.08	383.61
	W2N2	y=24625.74/(1 + 113.07e^–0.073x^)	0.996	46.73	82.81	64.77	36.08	449.42
	W2N3	y=25073.63/(1 + 110.70e^–0.074x^)	0.996	45.81	81.40	63.61	35.59	463.86
	W3N1	y=22549.49/(1 + 114.82e^–0.073x^)	0.994	46.94	83.02	64.98	36.08	411.53
	W3N2	y=25230.61/(1 + 98.41e^–0.073x^)	0.996	44.82	80.91	62.87	36.08	460.46
	W3N3	y=25948.89/(1 + 93.08e^–0.075x^)	0.996	42.89	78.01	60.45	35.12	486.54

W1, moderate water deficit (55%−65% of field water capacity, F_C_); W2, mild water deficit (65%−75% F_C_); W3, full irrigation (75%−85% F_C_). N1, low nitrogen rate (120 kg ha^–1^); N2, medium nitrogen rate (180 kg ha^–1^); N3, high nitrogen rate (240 kg ha^–1^). R^2^, determination coefficient; t_1_, the start time of the rapid growth period; t_2_, the termination time of the rapid growth period; t_max_, the occurrence time of the maximum cumulative rate; Δt, the duration of rapid growth; V_max_, the maximum accumulation rate.

### 3.3 Yield and 100-seed weight

**Figure 6:**
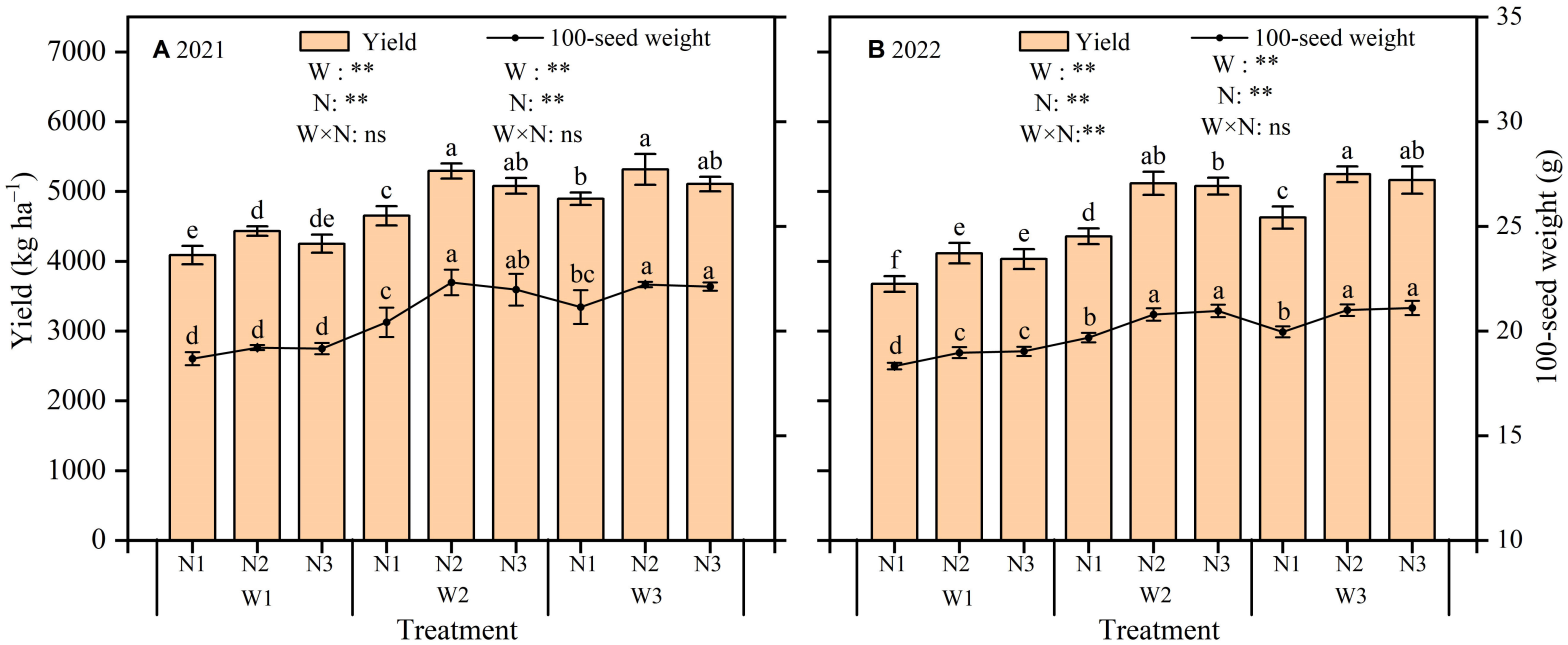
Effect of different irrigation and nitrogen treatments on sunflower yield and 100-seed weight in 2021 **(A)** and 2022 **(B)**. W1, moderate water deficit (55%−65% of field water capacity, F_C_); W2, mild water deficit (65%−75% F_C_); W3, full irrigation (75%−85% F_C_). N1, low nitrogen rate (120 kg ha^–1^); N2, medium nitrogen rate (180 kg ha^–1^); N3, high nitrogen rate (240 kg ha^–1^). Different lowercase letters above the error bar indicate significant differences between treatments in the same period at the P < 0.05 level. ** indicates a highly significant effect (P < 0.01); and ns indicates a non-significant effect (P > 0.05).

Different irrigation and N application treatments had highly significant (P < 0.01) effects on sunflower yield in both years (**Figure 6**). The interaction of water and N (W×N) had a highly significant effect on yield in 2022, while this effect was not significant (P > 0.05) in 2021. Yields across all treatments spanned from 4088.8 kg ha^–1^ to 5316.6 kg ha^–1^ in 2021 and from 3674.1 kg ha^–1^ to 5247.7 kg ha^–1^ in 2022. Specifically, yields from W3N3, W3N2, W2N2 and W2N3 treatments did not differ significantly in either year, yet these were significantly (P < 0.05) higher than other treatments. Under constant irrigation level, N2 obtained the highest yield of 5013.5 kg ha^–1^ in 2021 and 4826.4 kg ha^–1^ in 2022. While N3 demonstrated a slight yield reduction, this difference was insignificant compared to N2, suggesting that excess N fertilizer may not enhance yield and could potentially have a negative effect; whereas, N1 yielded significantly less, with reductions of 9.34% and 12.59% compared to N2 in both respective seasons. Besides, yields declined with increasing water deficit at constant N application. No significant yield differences were observed between W3 and W2. However, W3 yielded significantly more than W1, with increases of 19.92% in 2021 and 27.22% in 2022.

Irrigation and N application had a highly significant (P < 0.01) effect on 100-seed weight in both seasons. However, their interaction (W×N) had no significant (P > 0.05) effect on 100-seed weight (**Figure 6**). Specifically, 100-seed weight decreased with increasing water deficit. The lowest 100-seed weight was observed in W1, exhibiting reductions of 12.88% in 2021 and 9.24% in 2022 compared to W3. While W2 demonstrated a slight decrease in 100-seed weight compared to W3, this difference was not significant. No significant differences in 100-seed weight were observed between N2 and N3 under the same irrigation conditions. However, N1 demonstrated a significant reduction in 100-seed weight compared to N3, with decreases of 4.80% and 5.11% in the two years, respectively.

### 3.4 Water consumption and water and nitrogen use efficiency

Irrigation had a highly significant (P < 0.01) effect on ET in both 2021 and 2022. N application significantly (P < 0.05) affected ET in 2022, but not in 2021 (P > 0.05). W×N did not significantly affect ET in either year (**Table 4**). The total ET across the entire growing season for the different treatments ranged from 271.65 mm to 404.53 mm in 2021 and from 274.18 mm to 375.29 mm in 2022. The highest ET was observed in W3N3 and the lowest in W1N1. Specifically, ET exhibited a significant decrease with increasing water deficit. In 2021, the ET in W1 measured 275.34 mm, reflecting a reduction of 20.04% and 30.89% compared to W2 and W3, respectively. Similarly, in 2022, the ET in W1 measured 280.26 mm, indicating a reduction of 12.64% and 22.22% compared to W2 and W3, respectively.

**Table 4:** Effects of different irrigation and nitrogen application rates on water consumption and water and nitrogen use efficiency in 2021 and 2022.

Year	Treatment	ET (mm)	WUE (kg m^–3^)	IWUE (kg m^–3^)	NPFP (kg kg^–1^)
2021	W1N1	271.65 ± 18.91a	1.51 ± 0.04b	2.49 ± 0.26b	34.07 ± 1.10c
	W1N2	274.11 ± 16.40a	1.62 ± 0.05a	2.69 ± 0.13a	24.62 ± 0.39e
	W1N3	280.25 ± 10.12a	1.51 ± 0.06b	2.54 ± 0.18ab	17.72 ± 0.53g
	W2N1	324.60 ± 5.84b	1.43 ± 0.07c	2.19 ± 0.20d	38.78 ± 1.15b
	W2N2	330.81 ± 8.96b	1.60 ± 0.08a	2.46 ± 0.16b	29.40 ± 0.60d
	W2N3	336.15 ± 12.04b	1.51 ± 0.08b	2.34 ± 0.15c	21.17 ± 0.47f
	W3N1	392.14 ± 13.72c	1.25 ± 0.06e	1.79 ± 0.11e	40.79 ± 0.59a
	W3N2	398.61 ± 11.96c	1.33 ± 0.05d	1.94 ± 0.16e	29.53 ± 1.24d
	W3N3	404.53 ± 18.09c	1.26 ± 0.03e	1.84 ± 0.05e	21.28 ± 0.44f
Significance level	W	**	**	**	**
	N	ns	*	ns	**
	W×N	ns	ns	ns	*
2022	W1N1	274.18 ± 14.98d	1.34 ± 0.05c	2.96 ± 0.12a	30.62 ± 0.52c
	W1N2	280.62 ± 8.84d	1.47 ± 0.05b	3.15 ± 0.10a	22.86 ± 0.71e
	W1N3	285.97 ± 5.71d	1.41 ± 0.04bc	2.99 ± 0.15a	16.80 ± 0.30g
	W2N1	308.93 ± 11.13c	1.41 ± 0.03bc	2.41 ± 0.11c	36.30 ± 0.56b
	W2N2	315.29 ± 8.96c	1.62 ± 0.01a	2.70 ± 0.08b	28.42 ± 0.91d
	W2N3	322.85 ± 12.55c	1.57 ± 0.07a	2.63 ± 0.13b	21.15 ± 0.16f
	W3N1	347.07 ± 9.35b	1.33 ± 0.05c	1.85 ± 0.07d	38.55 ± 1.15a
	W3N2	358.63 ± 16.55ab	1.47 ± 0.06b	1.99± 0.05d	29.15 ± 0.33d
	W3N3	375.29 ± 13.01a	1.38 ± 0.02c	1.87 ± 0.10d	21.51 ± 0.89f
Significance level	W	**	**	**	**
	N	*	**	**	**
	W×N	ns	ns	ns	**

Data are presented as mean of three replicates ± standard deviation (n = 3). W1, moderate water deficit (55%−65% of field water capacity, F_C_); W2, mild water deficit (65%−75% F_C_); W3, full irrigation (75%−85% F_C_). N1, low nitrogen rate (120 kg ha^–1^); N2, medium nitrogen rate (180 N ha^–1^); N3, high nitrogen rate (240 kg ha^–1^). Different lowercase letters within a column indicate significant differences at the P < 0.05 level. * means a significant differences at the P<0.05 significance level, ** means a highly significant differences (P < 0.01); and ns means no significant differences (P > 0.05).

As shown in **Table 4**, WUE was significantly (P < 0.05) affected by irrigation and N application in both years. IWUE was significantly affected by both factors in 2022, while in 2021, N application had no significant (P > 0.05) effect on IWUE. In addition, W×N did not significantly affect either WUE or IWUE in both years. In 2021, both WUE and IWUE showed an increasing trend with increasing water deficit. Compared to W1, WUE and IWUE in W3 were lower by 17.09% and 28.17%, respectively. In 2022, W2 attained the highest WUE, surpassing W1 and W3 by 9.22% and 10.39%, respectively; whereas, W1 reached the highest IWUE, surpassing W2 and W3 by 17.71% and 59.50%, respectively. Besides, under the same level of water deficit, N2 consistently exhibited the highest WUE and IWUE in both seasons, with an increase of 8.64% and 10.43% respectively, compared to N1, which had the lowest WUE and IWUE.

Different irrigation and N application rates, along with their interactions (W×N), significantly (P < 0.05) influenced NPFP in both 2021 and 2022 (**Table 4**). In general, NPFP demonstrated an increasing trend with higher irrigation amounts but decreased with higher N application rates. Compared to W1, NPFP in W2 and W3 increased by 16.92% and 19.88% in 2021 and by 22.20% and 26.96% in 2022, respectively. In contrast, compared to N1, NPFP in N2 and N3 decreased by 26.47% and 47.06% in 2021 and by 23.73% and 43.62% in 2022, respectively.

### 3.5 Multi-objective optimization of irrigation and nitrogen application strategies

#### 3.5.1 Water and nitrogen regression models based on multiple objectives

**Figure 7:**
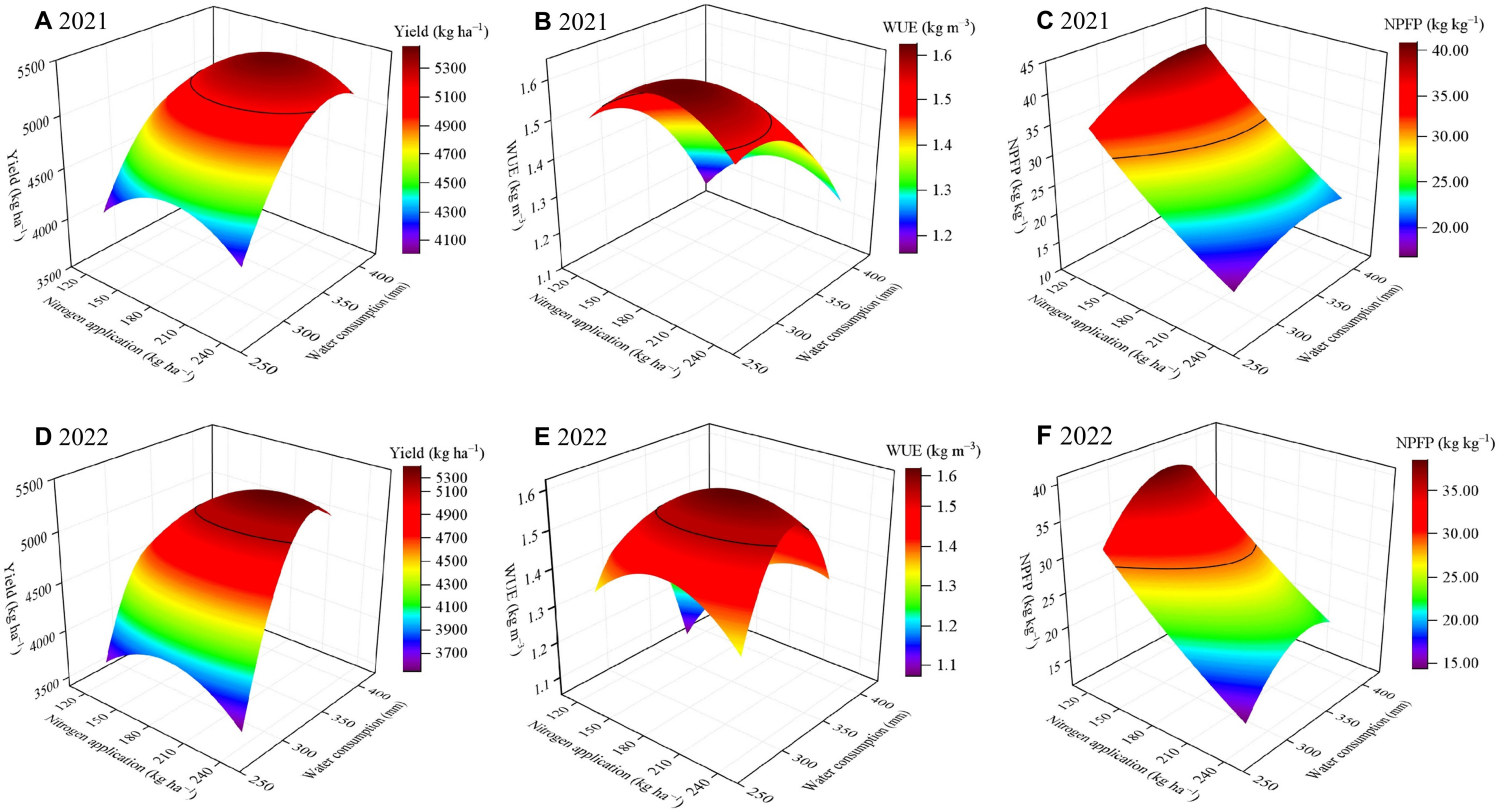
Relationships of sunflower yield, water use efficiency (WUE) and nitrogen partial factor productivity (NPFP) with water consumption (ET) and nitrogen application in 2021 **(A**–**C)** and 2022 **(D–F)**. The solid black lines in the figure represent 95% of the maximum yield and WUE and 75% of the maximum NPFP.

This study evaluated the effects of varying water and N application rates on sunflower production under film-drip irrigation. The objective was to identify optimal combinations for water conservation, fertilizer efficiency, high yield, and reduced environmental effect. Yield, WUE, and NPFP were selected as key indicators. The binary quadratic regression equations for the three target variables with ET and N application were developed according to the principle of least squares (**Table 5**; **Figure 7**). The resulting equations, equations, all significant with R^2^ values exceeding 0.96 in both 2021 and 2022, effectively indicated the interactive effects of water and N application on each indicator. These equations were then utilized to determine the ET and N application rates that maximized each indicator (**Table 5**). In 2021, maximum yield was achieved at 371.99 mm ET and 191.02 kg ha^–1^ N application. WUE peaked at 290.63 mm ET and 183.67 kg ha^–1^ N application, while NPFP reached its maximum at 382.84 mm ET and 120.00 kg ha^–1^ N application. In 2022, the highest yield occurred at 348.46 mm ET and 210.61 kg ha^–1^ N application. Maximum WUE was observed at 321.64 mm ET and 198.37 kg ha^–1^ N application, while NPFP was maximized at 348.46 mm ET and 120.00 kg ha^–1^ N application.

**Table 5:** Regression equations of sunflower yield, Water use efficiency (WUE) and nitrogen partial factor productivity (NPFP) with water consumption (ET) and nitrogen application rate in 2021 and 2022.

Year	Response variable	Regression equation (*y*)	R^2^	N and ET values at the maximum of the response variable		
				Max	N (kg ha^–1^)	ET (mm)
2021	Yield (kg ha^–1^)	*y=*–1.072×10^4^ + 70.917*x_1_* +30.557*x_2_* +0.018*x_1_x_2_*–0.100*x_1_* ^2^–0.097*x_2_* ^2^	0.966	5422.27	191.02	371.99
	WUE (kg m^–3^)	y*=*–1.082 + 0.013*x_1_* +0.010*x_2_* +4.953×10^–6^ *x_1_x_2_*–2.321×10^–5^ *x_1_* ^2^–2.974×10^–5^ *x_2_* ^2^	0.982	1.63	183.67	290.63
	PFPN (kg kg^–1^)	y*=*–17.083 + 0.433*x_1_*–0.220*x*_2_–1.3×10^–6^ *x_1_x_2_*–5.47×10^–4^ *x_1_* ^2 + ^3.10×10^–4^ *x_2_* ^2^	0.999	40.70	120.00	382.84
2022	Yield (kg ha^–1^)	y*=*–2.661×10^4^ + 177.137*x_1_* +10.162*x_2_* +0.088*x_1_x_2_*–0.280*x_1_* ^2^–0.097*x_2_* ^2^	0.992	5390.37	210.61	348.46
	WUE (kg m^–3^)	y*=*–6.741 + 0.049*x_1_* +0.004*x_2_* +2.636×10^–5^ *x_1_x_2_*–8.484×10^–5^ *x_1_* ^2^–3.129×10^–5^ *x_2_* ^2^	0.983	1.62	198.37	321.64
	PFPN (kg kg^–1^)	y*=*–113.438 + 1.009*x_1_*–0.228*x_2_*–2.735×10^–5^ *x_1_x_2_*–1.457×10^–3^ *x_1_* ^2 + ^2.35×10^–4^ *x_2_* ^2^	0.999	38.48	120.00	348.46

#### 3.5.2 Determination of optimal water and N intervals based on spatial analysis

**Figure 8:**
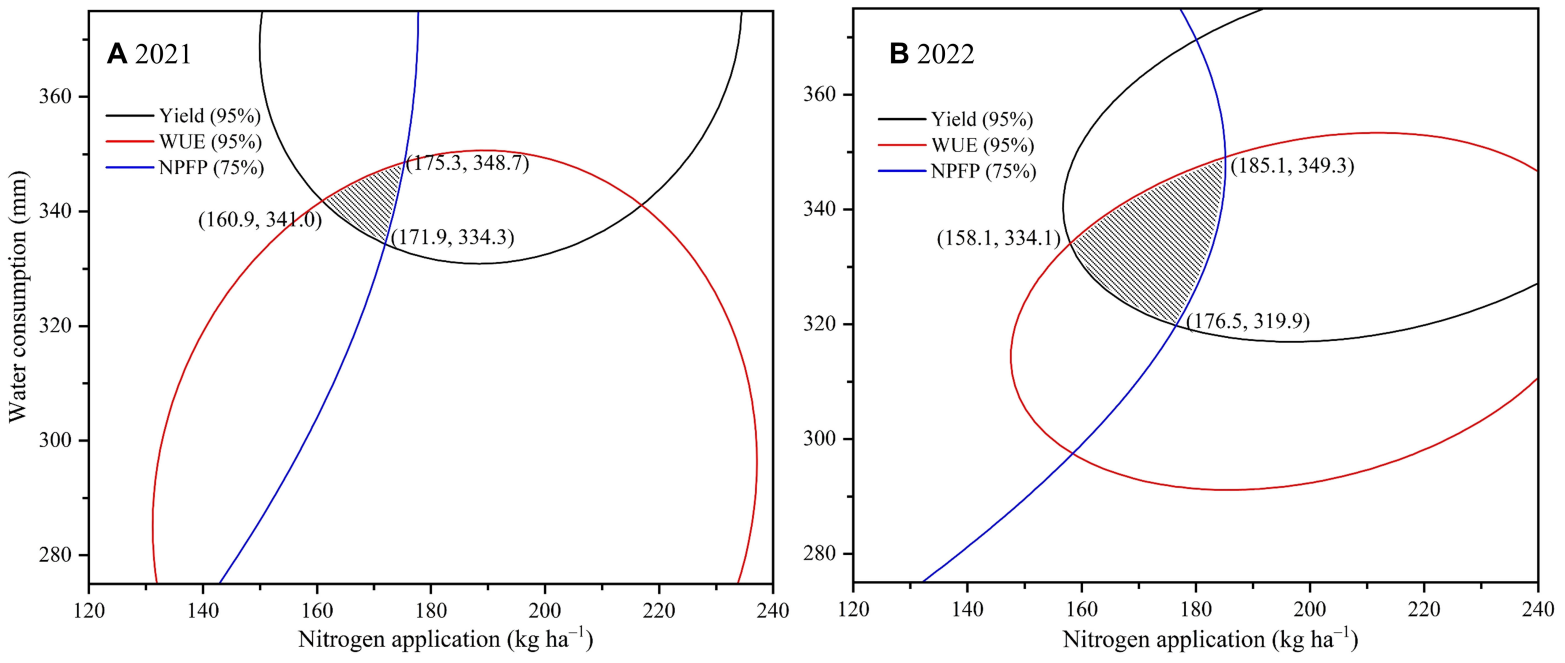
Comprehensive analysis based on yield and water use efficiency (WUE) and nitrogen partial factor productivity (NPFP) in 2021 **(A)** and 2022 **(B)**. The shaded areas indicate acceptable ranges where yield and WUE are greater than 95% of the maximum and NPFP is greater than 75% of the maximum.

The optimal water and N application strategy aims to maximize both water and N productivity while maintaining yield. However, achieving optimal sunflower yield, WUE and NPFP simultaneously proves challenging in practical agricultural settings. In the current study, the response surface analysis indicated a large interaction area between yield and WUE projections when both reached 95% of their maximum value (**Figure 7**); whereas, the projection of NPFP at 95% of its maximum demonstrated no interaction with the combined projection of yield and WUE at their respective 95% maxima. Therefore, in order to determine the optimal ET and N application rates that balance yield, WUE and NPFP, this study defined acceptable ranges as 95% of the maximum yield and WUE, and 75% of the maximum NPFP. The comprehensive analysis results were obtained by plane projection of these acceptable ranges (**Figure 8**). As illustrated, the ET intervals ensuring 95% of maximum sunflower yield and WUE, while maintaining NPFP at 75% of its maximum, were 334.3−348.7 mm in 2021 and 319.9−349.3 mm in 2022. Simultaneously, N application rates fell in the ranges of 160.9−175.3 kg ha^–1^ in 2021 and 158.1−185.1 kg ha^–1^ in 2022. Combining those two years’ findings suggests optimal ET and N application intervals for high yields and efficient utilization of water and N in sunflower in Hexi Oasis were 334.3−348.7 mm and 160.9−175.3 kg ha^–1^, respectively.

## 4 Discussion

### 4.1 Effect of different irrigation and nitrogen application on leaf area index and dry matter accumulation

LAI is a critical indicator for measuring the growth and development in crop populations. A higher LAI can give full play to the advantages of plant population, improve the photosynthetic capacity of the canopy, and promote the accumulation of photosynthetic products (Hikosaka, 2004). Previous studies have shown that crop LAI is susceptible to irrigation and fertilizer management practices (Zain et al., 2021). In this study, LAI in sunflower showed a significant increase followed by a slight increase at each growth stage with increasing irrigation and N application. Specifically, LAI was significantly lower (P < 0.05) in W1 compared to W2 and W3, with no significant difference (P > 0.05) between W2 and W3. Similarly, LAI was significantly lower in N1 compared to N2 and N3, with no significant difference between N2 and N3. These findings indicate that increasing water and N inputs in a reasonable range can significantly increase LAI. However, excessive applications of water and N do not lead to further significant increases in LAI. A similar finding was also reported by Yang et al. (2019), who observed a strong positive correlation between LAI and irrigation amount in sunflowers. Moreover, with increasing N fertilizer application, LAI in sunflower showed an increasing trend in the early and middle growth stages; whereas in the late growth stage, LAI showed an increasing and then decreasing trend.

As an important indicator of crop growth, DMA is closely related to yield formation (Wang et al., 2014b). Water and fertilizer management practices are used to regulate the final yield by influencing the crop DMA process (Guo et al., 2022). This study observed a positive correlation between irrigation levels and both DM and maximum DMA rates, aligning with observations made by Yang et al. (2019). This aligns with existing research indicating that increased water supply enhances both LAI and DMA (Gheysari et al., 2017), whereas water deficit can inhibit nutrient uptake and utilization by the root system, finally affecting both vegetative and reproductive growth (Fan et al., 2020). Furthermore, the results showed that under the same irrigation conditions, both DM and maximum DMA rates were significantly lower in N1 compared to N2 and N3. However, no significant differences were found between N2 and N3 for these parameters. This suggests that beyond a certain threshold, additional N application on may not significantly affect DMA. This finding is supported by Wu et al. (2023), who showed that N application increased DMA of each organ in sunflower, but excessive N application did not contribute to higher DMA compared to optimized N application. Similarly, Moraes et al. (2017) reported that N application significantly affected sunflower growth and development, but blindly increasing N fertilizer did not promote DMA.

### 4.2 Effect of different irrigation and nitrogen application on yield and water and nitrogen use efficiency

In agricultural production practices, water and N fertilizer management are essential measures to sustain plant growth and maintain stable yields (Chen et al., 2018). Many studies have demonstrated that in water-scarce regions, it is more beneficial to formulate a suitable irrigation and N application scheme based on the water and N requirements for crop growth rather than excessive supply (Li et al., 2022c; Si et al., 2020). In this study, the W3N2 treatment obtained the highest yield in both growing seasons. However, the difference was not significant (P>0.05) when compared to the yields of W3N3, W2N3, and W2N2. This suggests that a moderate reduction in water and N inputs, relative to full irrigation and high N fertilizer (W3N3), did not negatively affect sunflower yield formation. Consistent findings were obtained in several previous studies, which indicates that an appropriate supply of water and fertilizer can optimize resource access. Accordingly, this enhances photosynthetic capacity and carboxylation efficiency in plant leaves, finally leading to increased crop yield (Teixeira et al., 2014; Wang et al., 2015). Moreover, this study indicated a significant (P<0.05) reduction in sunflower yield under moderate water deficit and N treatments (W1N1, W1N2 and W1N3). This decline is attributed to the osmotic stress experienced by the plants due to the combined effects of water deficit and N application. This stress compromises the ability of the root system to absorb water and nutrients, finally resulting in yield reduction (Sheshbahreh et al., 2019).

Several previous studies have shown that appropriate irrigation and N application ratios can promote both water and nutrient availability, leading to significant improvements in water and N use efficiency (Dai et al., 2019; Xu et al., 2018). The results showed that under constant N application, reducing irrigation led to increased WUE and IWUE but decreased NPFP. This suggests that while appropriate water deficit improved water productivity, it limited the full utilization of fertilizer. In addition, under the same irrigation conditions, the medium nitrogen (N2) treatments resulted in the highest WUE and IWUE, with NPFP also reaching a high level. Similar results were obtained by Li et al. (2022b), who suggested that an optimal water and N management strategy should aim to align water and nutrient supply with crop uptake to better promote crop yield increases and efficient resource utilization.

### 4.3 Optimal water and nitrogen management strategies based on multi-objective optimization

Water scarcity and over-fertilization pose significant challenges to oasis farming in Northwest China. Accordingly, it is evident that enhancing water and fertilizer inputs to enhance crop yields is unsustainable. Therefore, irrigation and fertilizer management strategies that simultaneously optimize yield, water and fertilizer use efficiency are critical for ensuring sustainable agricultural production in the region. It is challenging to define optimal water and N application strategies through qualitative analysis alone, considering the inconsistent interaction effects of different water and N application rates on multiple production objectives. Numerous studies have employed multiple regression to establish the relationship between water and N inputs, yield, water and N use efficiency, utilizing spatial analysis to determine the optimal water and N application intervals (Gao et al., 2023; Wang et al., 2018). In this study, considering that the differences in rainfall between years would result in different water inputs to the cropland, we chose to build a binary quadratic regression model with ET and N application as explanatory variables and yield, WUE and NPFP as response variables. Subsequently, we defined acceptable conditions as 95% of the maximum values for yield and WUE, and 75% for NPFP. utilizing these thresholds, we solved for the water and N intervals that could simultaneously satisfy all three conditions. The results showed that multi-objective optimization and sustainable sunflower production in arid regions could be achieved with an ET range of 334.3 mm to 348.7 mm and a N application rate of 160.9 kg ha^–1^ to 175.3 kg ha^–1^. It is important to acknowledge the limitations of this study. Our analysis did not incorporate production objectives such as economic benefits and seed quality when determining optimal irrigation and N management strategies, potentially affecting the results. Future research should consider incorporating these additional production objectives into regression models to develop more accurate and comprehensive irrigation and N application management strategies.

## 5 Conclusions

This study indicated that different irrigation and N application rates had significant effects on sunflower LAI, DMA, ET, 100-seed weight, yield and water and N use efficiency. Increasing irrigation and N application enhanced LAI, DMA, ET 100-seed weight and yield. However, LAI, DMA, 100-seed weight and yield did not increase significantly when water and N inputs exceeded certain thresholds. Additionally, reducing irrigation significantly improved WUE and IWUE, while reducing N application significantly increased NPFP. Among all treatment combinations, full irrigation and high N treatment (W3N3) not only did not give the best yield benefit, but also resulted in inefficient use of water and nitrogen. Therefore, moderate reductions in irrigation and N application were more favorable for improving water and N productivity of sunflower in the arid environments. Optimization utilizing binary quadratic regression equations and spatial analysis indicated that a balance between water conservation, high yield, resource use efficiency and environmental sustainability could be achieved when ET was 334.3−348.7 mm and N application was 160.9−175.3 kg ha^–1^. Among the tested treatments, a mild water deficit combined with medium N rate (W2N2, 65%−75% F_C_ and 180 kg N ha^–1^) closely aligned with this optimal range. Therefore, the W2N2 treatment can be recommended as the optimal irrigation and N management strategy for sustainable sunflower production in the cold and arid Hexi Oasis region of northwestern China.

## Data Availability

The datasets presented in this study can be found in online repositories. The names of the repository/repositories and accession number(s) can be found in the article/supplementary material.
